# Changes in the flexion-relaxation response induced by hip extensor and erector spinae muscle fatigue

**DOI:** 10.1186/1471-2474-11-112

**Published:** 2010-06-04

**Authors:** Martin Descarreaux, Danik Lafond, Vincent Cantin

**Affiliations:** 1 Département de chiropratique, Université du Québec à Trois-Rivières, Trois-Rivières, G9A 5H7, Canada; 2 Département des sciences de l'activité physique, Université du Québec à Trois-Rivières, Trois-Rivières, G9A 5H7, Canada

## Abstract

**Background:**

The flexion-relaxation phenomenon (FRP) is defined by reduced lumbar erector spinae (ES) muscle myoelectric activity during full trunk flexion. The objectives of this study were to quantify the effect of hip and back extensor muscle fatigue on FRP parameters and lumbopelvic kinematics.

**Methods:**

Twenty-seven healthy adults performed flexion-extension tasks under 4 different experimental conditions: no fatigue/no load, no fatigue/load, fatigue/no load, and fatigue/load. Total flexion angle corresponding to the onset and cessation of myoelectric silence, hip flexion angle, lumbar flexion angle and maximal trunk flexion angle were compared across different experimental conditions by 2 × 2 (Load × Fatigue) repeated-measures ANOVA.

**Results:**

The angle corresponding to the ES onset of myoelectric silence was reduced after the fatigue task, and loading the spine decreased the lumbar contribution to motion compared to the hip during both flexion and extension. A relative increment of lumbar spine motion compared to pelvic motion was also observed in fatigue conditions.

**Conclusions:**

Previous results suggested that ES muscles, in a state of fatigue, are unable to provide sufficient segmental stabilization. The present findings indicate that, changes in lumbar-stabilizing mechanisms in the presence of muscle fatigue seem to be caused by modulation of lumbopelvic kinematics.

## Background

### Flexion-relaxation phenomenon (FRP)

The FRP is defined by reduced or silent myoelectric activity of the lumbar erector spinae (ES) muscle during full trunk flexion and is observed in healthy individuals [1]. It is believed to reflect the load-sharing interaction of the active and passive components of lumbopelvic stability [2]. During progressive trunk flexion, tension in the posterior ligaments and zygapophysial joints increases to a level where the active extension moment generated by the posterior muscles of the spine is no longer needed [1]. Olson et al. [3] reported that the FRP during trunk flexion from a standing position was not present during trunk flexion from a supine position and concluded that the gravitational load applied to the lumbar spine seems to be an important modulator of the flexion-relaxation response. Holm et al. [4] found that passive spinal elements play a critical role in sensorimotor control of the spine. They demonstrated that sensory feedback from passive viscoelastic structures of the spine is needed to regulate local muscle tension and lumbar spine stability. Therefore, activation and deactivation of the paraspinal muscles may be coordinated in such a manner that forces applied to the various structures are properly distributed and in such a pattern that loading on the motion segment is optimal regardless of the global spine position [4].

The onset and cessation of the flexion-relaxation response, corresponding to the beginning and the end of the myoelectric silence, can be influenced by several factors, such as trunk-loading [2], lumbopelvic posture [2,5], angular trunk velocity [6], task repetition [7] and muscular fatigue [7,8]. The effect of ES muscle fatigue, as an independent variable, on the FRP was assessed recently using the Sorensen protocol [8]. The results suggested that ES muscles, in a state of fatigue, may not be able to provide sufficient stabilization to the vertebral units, transferring load-sharing to passive structures earlier in trunk flexion. However, this study did not assess the potential changes in lumbopelvic rhythm following muscle fatigue.

### Hip extensor muscle role in lumbopelvic stabilization

Previous research, where hip extensor electromyography (EMG) was recorded, reported conflicting information regarding the hamstring muscles and a possible flexion-relaxation response [2,3,9]. Some studies have documented the myoelectric silence period of the hamstring muscles during full trunk flexion [3,9], whereas others failed to demonstrate a constant pattern of muscle activation [2]. Nevertheless, the functional role of the hip extensor muscles, including the gluteus and hamstring muscles, has been investigated extensively, and these muscles seem to be actively involved in lower back stabilization as well as in lumbopelvic rhythm [10-13]. For instance, van Wingerden et al. [14] showed that the biceps femoris and gluteus maximus muscles can increase sacroiliac joint stabilization through their specific and massive attachments to the sacrotuberous ligament. Their results also indicated that the ES and hip extensor muscles clearly interact to provide lumbopelvic stabilization. During trunk flexion, the pelvis rotates freely anteriorly. As tension and stiffness increase in the hamstrings, pelvic rotation decreases, consequently generating more tension in the muscles and thoracolumbar fascia. Finally, additional trunk flexion will yield a reduction in the ratio of active to passive extensor moments to the point of full flexion-relaxation of the ES [15]. Altogether, it seems that hip extensor and ES muscles are both anatomically and functionally linked during the trunk flexion task.

### Objectives

Previous experiments suggest that ES muscle fatigue modifies FRP parameters. However, it has not been investigated whether such effects are due to decreased spinal stabilization from active structures or are the result of modified lumbopelvic rhythm. Therefore, the objectives of this study were 2-fold: [1] to quantify the effect of hip and back extensor muscle fatigue on FRP parameters, and [2] to determine if such changes are triggered by modifications of lumbopelvic dynamics. We hypothesized that hip extensor muscle fatigue will lead to reduced pelvic flexion and an increased myolectric silence period (FRP) during a flexion-extension task in association with changes in lumbopelvic rhythm.

## Methods

### Participants

Twenty-seven healthy adults (age: 23.6 ± 2.1 years, weight: 64.2 ± 12.8 kg, height: 1.69 ± 0.07 m), 13 men and 14 women with no history of low back pain (LBP), participated in this study. All study subjects gave their informed, written consent according to a protocol approved by the Université du Québec à Trois-Rivières (Canada) Ethics Committee. Participants with present or past LBP or thoracic pain, spinal trauma or surgery were excluded from the experiment.

### Experimental protocol

The experimental task involved 12 cycles of trunk flexion/extension movement with a 30-s rest period between each cycle. The participants were required to bend forward as far as possible during each 5-s movement period (flexion phase). They were then instructed to hold the fully-flexed position for 3 s. The extension phase lasted 5 s and enabled the participants to return to the initial upright standing position. A movement time of 5 s was chosen to replicate the experimental protocol used in previous studies [8,16]. An auditory cue served to standardize the movement phase duration and then to control trunk velocity. Instructions followed by a demonstration of the flexion-extension task were given to the study participants prior to the experimental trials. Sufficient practice (3 to 5 trials followed by rest) was allowed to ensure that the participants performed the task correctly prior to data acquisition.

**Figure 1:**
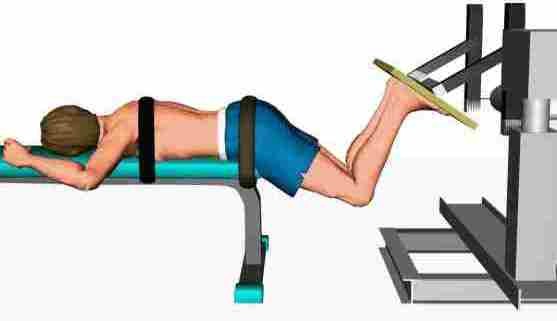
**Fatigue task**. Fatigue task during which participants were instructed to push upwards with both legs with an isometric contraction set at 60% of their MVC force (peak value from the two MVC trials).

The study subjects underwent blocks of 3 trials of the flexion-extension task under 4 different experimental conditions: **(1) **no fatigue/no load, **(2) **no fatigue/load, **(3) **fatigue/no load, and **(4) **fatigue/load. The "non-fatigue" conditions were always presented before the "fatigue" conditions. However, the "load" condition was randomized across participants. For the loading condition, a 12-kg disk was held with arms crossed on the shoulders. Hip and back extensor muscle fatigue was induced by sustained isometric contractions. Firstly, two maximal voluntary contractions (MVC) were performed using 5 s ramp isometric hip extensor contraction efforts. Briefly, each subject lay prone with the iliac crest aligned with the edge of the table, knees bent to 90° and a hip flexion angle of 60° (Figure 1).

The trunk was fixed to the table by stabilizing bands at the thoracic spine and pelvic region. The participants were asked and verbally encouraged to push a board upwards with both legs at the same time. The feet were placed flat at the pelvis width on the dynamometer plate. During the fatiguing task, subjects were asked to reproduce the same isometric contraction at 60% of their MVC force (peak value from the two MVC trials) and to maintain the contraction (using visual feedback) until exhaustion. The fatiguing test was stop by the investigator if the subject failed twice to maintain the force level between 55-65% MVC. Verbal encouragement was given through the test.

### Instrumentation

Kinematics data were collected by a motion analysis system (Optotrak Certus, Northern Digital, Waterloo, Ontario, Canada). Light-emitting diodes (LEDs) were positioned on the right side and back of each subject on the following anatomical landmarks: a) lateral malleolus, b) lateral part of the knee, c) greater trochanter, d) posterior superior iliac spine (PSIS), e) middle of the iliac crest, f) L2 spine, g) L1 spine, and h) T10 spine. The kinematics data were recorded at 100 Hz and low-pass filtered by a dual-pass, fourth-order Butterworth filter with a cut-off frequency at 5 Hz.

Surface EMG data were collected by bipolar disposable Ag-AgCl electrodes (Bortec Biomedical, Alberta, Canada) applied bilaterally over the ES at the L2-L3 level (≈ 2 cm from the midline), over the gluteus maximus at the mid-point between the middle of the sacrum and the greater trochanter, and over the biceps femoris at the mid-point between the fibula head and the ischiatic tuberosity. Electrodes were positioned parallel to the muscle fibre orientation with a centre-to-centre distance of 2.5 cm. Skin impedance was reduced by: 1) shaving excess body hair, if necessary, 2) gently abrading the skin with fine-grade sandpaper and wiping the skin with alcohol swabs. A reference electrode was placed over the left patella. EMG signals were differentially amplified (AMT-8, common mode rejection ratio of 115 dB at 60 Hz, input impedance of 10 GW; 12-bit A/D converter) and sampled at 900 Hz. The EMG data were digitally filtered with a zero phase lag, bi-directional, 10 to 450 Hz bandpass fourth-order Butterworth filter.

### Data analyses

**Figure 2:**
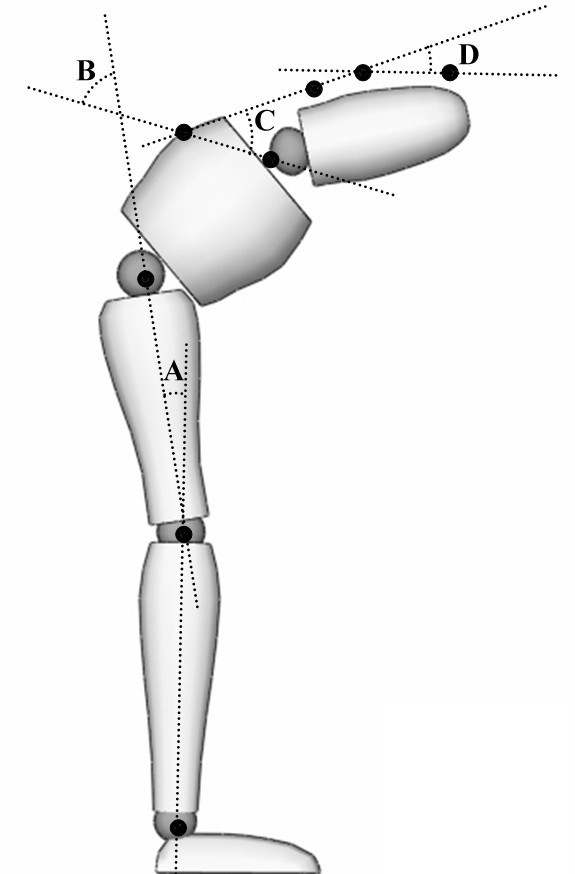
**Angles and LEDs positioning**. Illustration of LEDs positioning and the thoracic (D), lumbar (C), hip (B) and knee angles (A).

Two adjacent LEDs were used to form a vector and the angles between vectors served to quantify thoracic, lumbar spine and pelvic motion, as illustrated in Figure 2. Thoracic motion was defined as the angle between the T10-L1 and L1-L2 vectors. Lumbar spine motion was obtained by the angle between the L1-L2 and PSIS-iliac crest vectors. Hip motion was determined by the angle between the PSIS-iliac crest and greater trochanter-knee vectors. Total trunk flexion angle was calculated as the sum of thoracic, lumbar spine and hip angles. The lumbar spine and hip angles were then used to calculate the lumbar/hip (L/H) ratio. Subsequently, the total flexion and extension angles were divided into quartiles, and the L/H ratio was associated with each quartile (Q1-Q4).

The rectified EMG signals and kinematics data (total trunk flexion angle) were plotted to determine the total trunk angle corresponding to EMG cessation during the flexion phase and the total trunk angle of EMG onset during the extension phase. EMG cessation and onset were quantified by visual inspection of the rectified EMG signal. Modulations of the EMG signal amplitude of each muscle during all movement phases were calculated by root mean square (RMS). Muscle fatigue during the fatiguing protocol was assessed through power spectral analysis of the EMG data (fast Fourrier transform). The rate of decline of median frequency with time (MedF/time slope) was calculated to confirm that muscle fatigue was induced in the targeted muscles.

### Statistical analyses

Total flexion angle corresponding to the onset and cessation of myoelectric silence, hip flexion angle, lumbar flexion angle and maximal trunk flexion angle were compared across different experimental conditions by 2 × 2 (Load × Fatigue) repeated-measures ANOVA. RMS values of the ES muscles through all movement phases (Q1-Q4) were analyzed according to the same experimental plan. The level of statistical significance was set at p < 0.05 for all analyses.

## Results

Mean time until exhaustion during the fatiguing protocol was 80.2 ± 23.4 s. The mean rate of decline in MedF/time was -0.208 ± .197 Hz/s for the hamstrings, -0.105 ± .111 Hz/s for the gluteus maximus, and -0.244 ± .106 Hz/s for the paraspinal muscles, indicating that muscular fatigue was induced prior to the FRP tasks. Overall, 5 participants did not present hamstring fatigue while 3 participants did not present hamstring fatigue.

### Flexion-relaxation phenomenon

Statistical analyses yielded a significant muscular fatigue effect on FRP onset angles for both the right and left ES muscles (Figure 2). The angle corresponding to the onset of myoelectric silence was significantly reduced at the right ES muscle (95.1 ± 3.9° vs 90.9 ± 3.9°) and the left ES muscle (92.7 ± 3.4° vs 89.1 ± 3.7°) after the fatiguing protocol. During loading conditions, fatigue conditions did not affect the FRP cessation angle. A significant increase in FRP onset angle, cessation angle and total trunk flexion angle was also observed during loading conditions (Table 1).

**Table 1:** Mean (standard error) FRP onset, FRP cessation and total trunk flexion angles (°) during non-loading and loading conditions

		Mean	SE	95% CI -	95% CI +
**Onset 1**	No load	89.9	3.6	97.5	82.3
	Load	97.0	3.7	104.8	89.2

**Cessation 1**	No load	102.2	2.6	107.6	96.8
	Load	107.0	3.1	113.4	100.4

**Onset 2**	No load	88.5	3.5	95.7	81.1
	Load	95.7	3.5	103.0	88.3

**Cessation 2**	No load	101.1	2.9	107.2	95.0
	Load	107.0	3.2	113.6	100.2

**ROM total**	No load	119.8	2.8	113.9	125.7
	Load	123.6	2.8	117.6	129.5

### Lumbopelvic kinematics and related ES activity

**Figure 3:**
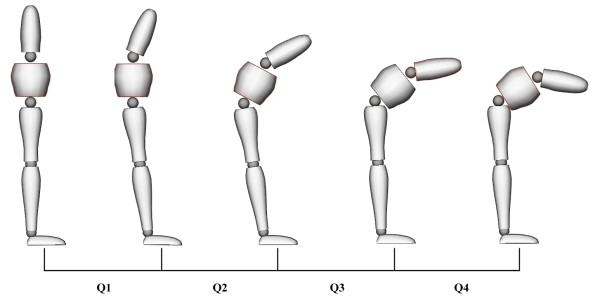
*L/H ratio, loading, fatigue and interaction effects during flexion (Q1-Q4). Data are presented for the (1) no load-no fatigue (2) no load-fatigue (3) load-no fatigue and (4) load-fatigue conditions.

**Figure 4:**
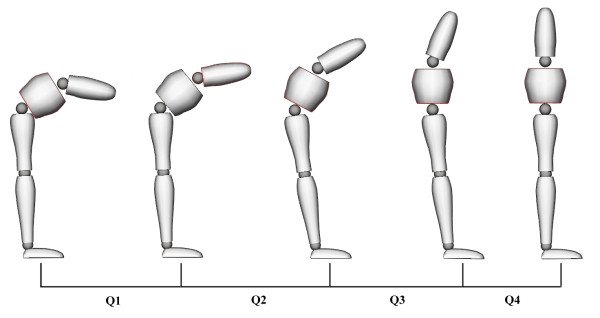
*L/H ratio, loading, fatigue and interaction effects during extension (Q1-Q4). Data are presented for the (1) no load-no fatigue (2) no load-fatigue (3) load-no fatigue and (4) load-fatigue conditions.

Loading the spine significantly decreased (p < 0.001) the L/H ratio during Q1 and Q2 of the flexion phase and during Q2 of the extension phase. Reduction of the L/H ratio indicated that relative flexion of pelvic motion increased in comparison to lumbar angle during the loading conditions. Fatigue conditions yielded a significant rise (p < 0.05) in the L/H ratio during Q2 of the flexion phase and during Q4 of the extension phase, indicating a relative augmentation of lumbar spine motion compared to pelvic motion. The following figures and tables present the L/H ratio as well as loading and fatigue effects through all quartiles of the flexion (Figure 3 and Table 2) and extension (Figure 4 and Table 3) phases of movement.

**Table 2:** L/H ratio, loading effects and fatigue effects during flexion (Q1-Q4)

Flexion L/H ratio	0-25	25-50	50-75	75-100
1	1.34 (0.11)	1.08 (0.07)	0.96 (0.07)	0.42 (0.05)
2	1.41 (0.14)	1.20 (0.09)	1.01 (0.08)	0.45 (0.04)
3	1.06 (0.08)	0.93 (0.06)	0.96 (0.08)	0.49 (0.05)
4	1.18 (0.12)	0.99 (0.07)	0.94 (0.07)	0.51 (0.05)

ANOVA main and interaction effects				

Load	**p < 0.001**	**p < 0.001**	0.4040	0.0679
Fatigue	0.1542	**p < 0.05**	0.7159	0.4162
Load × Fatigue	0.7412	0.2027	0.1523	0.7728

**Table 3:** L/H ratio, loading effects and fatigue effects during extension (Q1-Q4)

Extension Ratio L/H	0-25	25-50	50-75	75-100
1	0.51 (0.06)	0.94 (0.07)	1.09 (0.07)	1.34 (0.13)
2	0.53 (0.07)	0.94 (0.07)	1.11 (0.07)	1.48 (0.14)
3	0.56 (0.07)	0.80 (0.06)	1.04 (0.08)	1.27 (0.12)
4	0.42 (0.06)	0.72 (0.07)	1.06 (0.07)	1.67 (0.15)

ANOVA main and interaction effects				

Load	0.4563	**p < 0.001**	0.1850	0.5380
Fatigue	0.3346	0.2232	0.6592	**p < 0.001**
Load × Fatigue	**p < 0.01**	0.1426	0.7765	**p < 0.05**

Loading the spine significantly increased (*p *< 0.05) RMS values of the ES muscles through all quartiles (Q1-Q4) of the flexion and extension phases. Similar significant increments of RMS values during loading were also observed during the full flexion phase (relaxation) (*p *< 0.05). Fatigue conditions, however, did not modify RMS values in any of the movement phases (*p *> 0.05).

## Discussion

The results of the present study indicate that fatigue of the hip extensor and ES muscles modifies lumbopelvic rhythm and, consequently, FRP parameters. In healthy participants, fatigue of these muscles led to reduced hip flexion angle (increased L/H ratio) and decreased FRP onset angle. However, fatigue did not modify EMG activity during the flexion-extension cycle. Previous data [8] suggested that ES muscles, in a state of fatigue, were unable to provide sufficient stabilization to the vertebral units, transferring load-sharing to passive structures earlier in trunk flexion. Alternatively, the present findings indicate that lumbar-stabilizing mechanisms, when hip extensor (gluteus or hamstring muscles) and ES muscles are fatigued, may remain unchanged. An apparent earlier onset of myoelectric silence in total trunk flexion angle seems to be caused by a change in lumbopelvic dynamics. Lumbopelvic dynamics during a flexion-extension cycle have been studied in the past [11,13,17], and it has been shown several times that the first half of the flexion phase occurs primarily at lumbar spine segments with the pelvis remaining relatively fixed, whereas the final half of flexion is accomplished primarily by forward pelvic rotation. A reverse mechanism has also been described during extension from a flexed posture where initial movement is achieved primarily at the hip level with an increasing contribution from the lumbar spine in the later stages of extension [18]. In the present experiment, it is possible that fatigue probably augmented stiffness in the hip extensor muscles. Heightened passive tension in the hamstring muscles has been suggested to limit pelvic movement during flexion and to facilitate it in early extension [18]. Hashemirad et al. [19] recently reported that subjects presenting less general flexibility (toe-touch test) showed decreased FRP onset and cessation angles. They suggested that when sufficient passive tension values are reached (monitored by spine mechanoreceptors), the central nervous system deactivates the active controlling element of movement (ES). Alternatively, recent studies showed that immediately following static and cyclic loading of the spine, laxity can develop in the associated viscoelastic structures without neuromuscular compensation mechanisms [20,21]. In the present experiment, such responses to loading (fatigue task) may have occurred and may have led to increased displacement and tension neutral zone and subsequent changes in lumbopelvic dynamics. Whether the modulating effect of hip and lumbar extensor muscle fatigue derives from changes in soft tissue mechanical properties or from alterations in neuromuscular strategies remains to be determined. Nevertheless, although it has often been suggested that hip extensor muscles can generate tension in passive lumbopelvic structures, such as the sacrotuberous ligament and the thoracolumbar fascia [22], the results of the present study indicate that hip and lumbar extensor muscle fatigue may challenge spinal stability requirements by changing lumbopelvic dynamics. Repeated trunk flexion and extension as well as lifting tasks have been previously targeted as potential causes of work related low back pain [21]. Therefore, repeated trunk movement or sustained static posture leading to muscle fatigue of back or hip extensor muscles may alter usual spinal loading and stability mechanisms therefore putting, the lumbar spine at risk of injury or reinjury.

Finally, the addition of a load anterior to the trunk modified the FRP response. FRP onset and cessations angles were increased in loading conditions. Several authors have reported a similar effect of load positioned either anteriorly or posteriorly to the trunk [2,8,23]. Such a decrease in the EMG silence period during flexion reflects the need for additional muscular contraction to counteract the increased flexion moment generated by the load, but can also be explained by the increment of total flexion angle during loading conditions. This augmentation of FRP onset and cessation angles was accompanied by heightened ES muscle activity through all phases of the flexion/extension cycle. Increased loading of the spine also led to a greater contribution of the hip in both flexion and extension movements, again illustrating a change in lumbopelvic dynamics when stability requirements are modified.

### Limitations

At this stage, we cannot exclude the possibility that the deep ES muscles increased their contraction level to compensate for a potential lack of lumbopelvic stability after hip and lumbar extensor muscle fatigue. Studies of the deep muscle contribution and combined effects of muscle fatigue and modification of passive structure mechanical properties are warranted to better understand the stability mechanisms underlying the FRP response. Moreover, studies involving clinical populations are needed to assess whether the changes observed in this experiment have any link to lower back and pelvic conditions.

## Conclusion

Spinal stability may be compromised by insufficient muscle force and inappropriate neuromuscular activation. Therefore, muscular fatigue of the hip extensor and ES muscles may temporarily modify lumbopelvic dynamics. The clinical implications of changes in lumbopelvic movement and whether or not these changes increase the risk of re-injury have yet to be studied.

## Competing interests

The authors declare that they have no competing interests.

## Authors' contributions

MD participated in study design, experimentation, data analysis and manuscript writing. DL and VC helped in the experimentation, data analysis and manuscript drafting. All authors read and approved the final manuscript.

## Pre-publication history

The pre-publication history for this paper can be accessed here:

http://www.biomedcentral.com/1471-2474/11/112/prepub
